# CRISPR-LbCpf1 prevents choroidal neovascularization in a mouse model of age-related macular degeneration

**DOI:** 10.1038/s41467-018-04175-y

**Published:** 2018-05-10

**Authors:** Taeyoung Koo, Sung Wook Park, Dong Hyun Jo, Daesik Kim, Jin Hyoung Kim, Hee-Yeon Cho, Jeungeun Kim, Jeong Hun Kim, Jin-Soo Kim

**Affiliations:** 1 Center for Genome Engineering, Institute for Basic Science, Seoul, 151-747 Republic of Korea; 2 Department of Basic Science, University of Science and Technology, Daejeon, 34113 Republic of Korea; 3 FARB Laboratory, Biomedical Research Institute, Seoul National University Hospital, Seoul, 03082 Republic of Korea; 4 Department of Biomedical Sciences, Seoul National University College of Medicine, Seoul, Republic of Korea; 5 Department of Ophthalmology, Seoul National University College of Medicine, Seoul, 03080 Republic of Korea; 6 Department of Chemistry, Seoul National University, Seoul, 151-747 South Korea

## Abstract

LbCpf1, derived from *Lachnospiraceae bacterium* ND2006, is a CRISPR RNA-guided endonuclease and holds promise for therapeutic applications. Here we show that LbCpf1 can be used for therapeutic gene editing in a mouse model of age-related macular degeneration (AMD). The intravitreal delivery of LbCpf1, targeted to two angiogenesis-associated genes encoding vascular endothelial growth factor A (*Vegfa*) and hypoxia inducing factor 1a (*Hif1a*), using adeno-associated virus, led to efficient gene disruption with no apparent off-target effects in the retina and retinal pigment epithelium (RPE) cells. Importantly, LbCpf1 targeted to *Vegfa* or *Hif1a* in RPE cells reduced the area of laser-induced choroidal neovascularization as efficiently as aflibercept, an anti-VEGF drug currently used in the clinic, without inducing cone dysfunction. Unlike aflibercept, LbCpf1 targeted to *Vegfa* or *Hif1a* achieved a long-term therapeutic effect on CNV, potentially avoiding repetitive injections. Taken together, these results indicate that LbCpf1-mediated in vivo genome editing to ablate pathologic angiogenesis provides an effective strategy for the treatment of AMD and other neovascularization-associated diseases.

## Abstract (web summary)

The CRISPR endonuclease LbCpf1 is reported to have greater efficiency and specificity than Cas9. Here, the authors use LbCpf1 to target the angiogenesis-related genes VEGF and HIF1a, and show that delivery of the nuclease using AAV9 is effective in mouse models of macular degeneration.

## Introduction

Wet age-related macular degeneration (AMD), a neovascular disease that leads to vision loss in the center of the visual field, occurs most commonly in individuals over the age of 50^1^. Several angiogenic growth factors in the retina have been implicated, including vascular endothelial growth factor (VEGF)^2^. The advent of anti-VEGF agents (e.g., aflibercept, bevacizumab, and ranibizumab) has led to significantly reduced retinal and choroidal neovascularizations (CNV) in the clinic^3,4^. However, currently used anti-VEGF therapies require frequent repetitive injections over time to sustain the therapeutic effect on ocular neovascularization^5–7^. Therefore, it is ideal to suppress pathologic angiogenesis with a single treatment over the long-term.

The type II CRISPR (clustered regularly interspaced short palindromic repeats) system, an adaptive immune system used by bacteria and archaea to defend against viral infection^8^, has been repurposed for site-specific genome modification^9–12^ and become a promising approach for the treatment of diverse genetic or non-genetic disorders^13–16^. The Cas9 protein (SpCas9), derived from *Streptococcus pyogenes*, in combination with a small-guide RNA (sgRNA), a synthetic fusion of CRISPR RNA (crRNA) and transactivating crRNA (tracrRNA)^17^, recognizes and cleaves a target locus, generating a blunt-ended double-stranded break (DSB) three nucleotides upstream of a protospacer adjacent motif (PAM). However, the relatively large size of the SpCas9-coding sequence (4.10 kbp encoding 1,368 amino acids) makes it difficult or impossible to package the system with a sgRNA expression cassette into an adeno-associated virus (AAV). Furthermore, concerns remain about SpCas9 off-target nuclease activity. These issues have accelerated efforts to develop alternative genome editing tools.

A recently reported RNA-guided endonuclease, CRISPR from *Prevoltella* and *Francisella* 1 (Cpf1), is derived from a type V (class II) CRISPR system and differs from Cas9 in several key respects^18^. Cpf1 is guided by a single crRNA, without the need for a tracrRNA^18^. In contrast to Cas9, Cpf1 exhibits ribonuclease activity, enabling it to process its precursor crRNA into mature crRNAs^19^. Cpf1 recognizes a T-rich PAM (5′-TTTV-3′) at the 5′- end of a protospacer; DNA cleavage generates a staggered DSB distal to the PAM site^20^. It has been reported that LbCpf1 from *Lachnospiraceae* *bacterium* ND 2006 and AsCpf1 from *Acidaminococcus sp*. BV3L6 induce DNA modifications in human cells with equal or greater efficiency than do Cas9 orthologues, including SaCas9, StCas9, and NmCas9 from *Staphylococcus aureus*, *Streptococcus thermophilus*, and *Neisseria meningitidis*, respectively^21^. In addition, there are several reports demonstrating that LbCpf1 exhibits a higher genome-wide specificity compared to SpCas9^22,23^.

To test whether LbCpf1 can be used as a gene therapy tool for the treatment of retinal diseases with the advantage of a smaller gene size (3.7 kbp encoding 1,299 amino acids) compared to SpCas9, we packaged sequences encoding LbCpf1 and its crRNA (total transgene cassette size; 4.7 kbp) in all-in-one AAV vector and delivered the resulting AAV into the mouse retina via intravitreal injection. We found that insertions and deletions (indels), a measure of Cpf1 activity, were induced at high frequencies in the *Vegfa* and *Hif1a* genes with target specificity. In particular, *Vegfa* or *Hif1a* disruption led to a long-term reduction of the area of laser-induced CNV without causing cone dysfunction. These findings suggest that Cpf1 has great potential as an in vivo genome editing therapy for the treatment of angiogenesis-related diseases.

## Results

### CRISPR-LbCpf1-mediated gene editing in vitro and in vivo

**Fig. 1:**
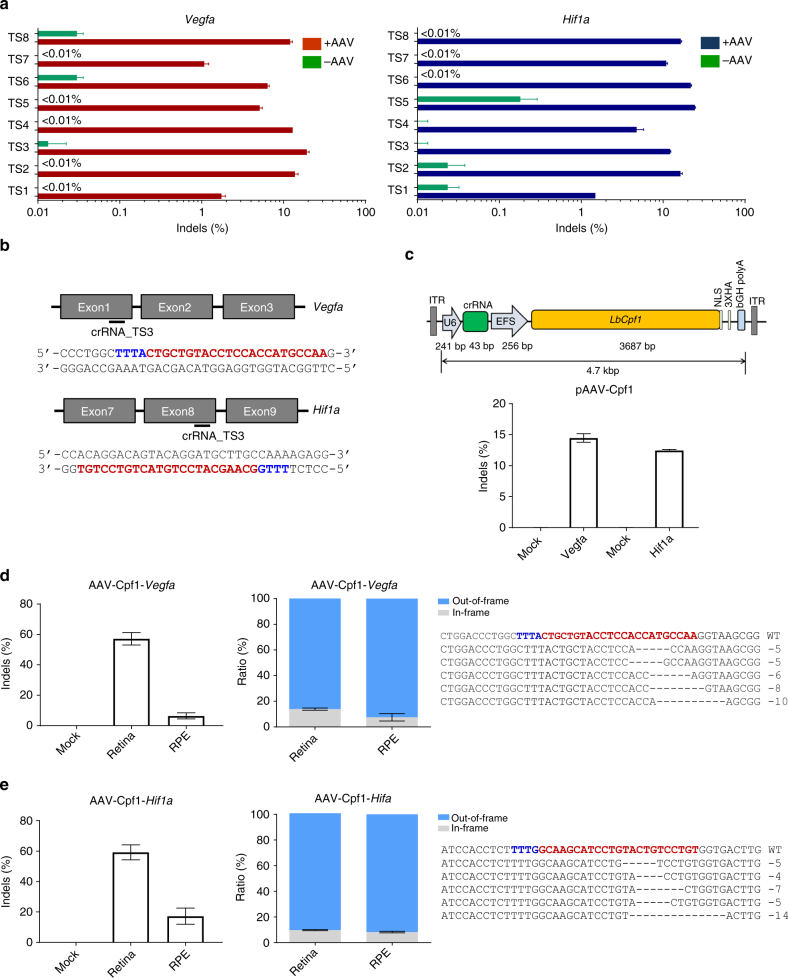
Genome editing with LbCpf1 in vitro and in vivo. **a** Mutation frequencies at the *Vegfa* and *Hif1a* target sites in C2C12 cells co-transfected with various crRNAs and LbCpf1 genome editing efficiencies were examined by deep sequencing using genome DNA isolated from cells after 48 h of transfection. Error bars indicate s.e.m. (*n* = 3). **b** The Cpf1 target sequences in the *Vegfa* and *Hif1a* genes. The PAM sequence and the crRNA target sequence (TS3) are shown in blue and red, respectively. **c** All-in-one AAV vector plasmids encoding Cpf1 and its crRNA (TS3) and its mutation frequencies at the *Vegfa* and *Hif1a* target sites in C2C12 cells transfected with pAAV-Cpf1-*Vegfa* or *Hif1a* plasmids 48 h post-transfection. **d**,**e** Indel frequencies and mutant sequences presented with two fractions, in-frame versus out-of-frame indels at the *Vegfa* (**d**) or *Hif1a* (**e**) target site in the retina and RPE cells using deep sequencing 6 weeks post-intravitreal injection of AAV-Cpf1-*Vegfa* or AAV-Cpf1-*Hif1a*. The PAM sequence and the crRNA target sequence (TS3) are shown in blue and red, respectively. Error bars indicate s.e.m. (*n* = 4)

To evaluate LbCpf1-mediated genome editing of angiogenesis-associated genes, we designed several crRNAs to target *Vegfa* or *Hif1a* exons. Cpf1 nucleases complexed with these crRNAs induced indels at frequencies that ranged from 1.1 ± 0.1% to 24.5 ± 0.4% in C2C12 mouse myotubes (Fig. 1a; Supplementary Table 1). We selected the TS3 and TS3 crRNAs, which target the exon 1 of *Vegfa* and exon 8 of *Hif1a* genes, respectively (Fig. 1b), because they both resulted in high ratio of out-of-frame indels as a representative mutation pattern with high efficiencies (Fig. 1a; Supplementary Fig. 1).

To investigate the genome editing efficiency of LbCpf1 in the mouse retina, we expressed Cpf1 with *Vegfa* (TS3) or *Hif1a* (TS3) specific crRNA in a single AAV vector plasmid (Fig. 1c). Cpf1 induced indels at frequencies of 14.5 ± 0.7% and 12.5 ± 0.2%, respectively in C2C12 mouse myotubes (Fig. 1c). Then, we packaged sequences encoding LbCpf1 and *Vegfa*- or *Hif1a*-specific crRNA into an AAV serotype 9 vector. The resulting viruses were administered into the mouse eye via intravitreal injection. AAV2/9 expressing *Vegfa*-specific LbCpf1 (AAV-Cpf1-*Vegfa*) induced indels with frequencies of 57.2 ± 4.1% and 6.5 ± 2% in the retina and RPE, respectively, 6 weeks post-injection (Fig. 1d). Analysis of indel-bearing sequences showed that 86 ± 0.9% and 93 ± 2.9% carried out-of-frame mutations induced by LbCpf1 in the retina and RPE, respectively (Fig. 1d; Supplementary Fig. 2). AAV2/9 expressing *Hif1a*-specific LbCpf1 (AAV-Cpf1-*Hif1a*) induced indels with frequencies of 59.2 ± 4.9% and 17.2 ± 5.3% in the retina and RPE, respectively, 6 weeks post-injection (Fig. 1e). Analysis of indel-bearing sequences showed that 90 ± 0.4% and 92 ± 0.6% carried out-of-frame mutations in the retina and RPE, respectively (Fig. 1e; Supplementary Fig. 2). As expected, an HA tag conjugated to the C-terminus of LbCpf1 was detected in both retina and RPE cells, indicating that LbCpf1 was successfully expressed (Supplementary Figs. 3, 4).

We compared indel frequencies at the *Hif1a* target site at 4 and 6 weeks after intravitreal injection of AAV9-Cpf1-*Hif1a*. We observed an increase in indel frequencies from 3.7 ± 1.6% to 17.2 ± 5.3% in RPE cells but did not observe an increase in the retina (Supplementary Fig. 5).

### Target specificity of LbCpf1 in the mouse retina

**Fig. 2:**
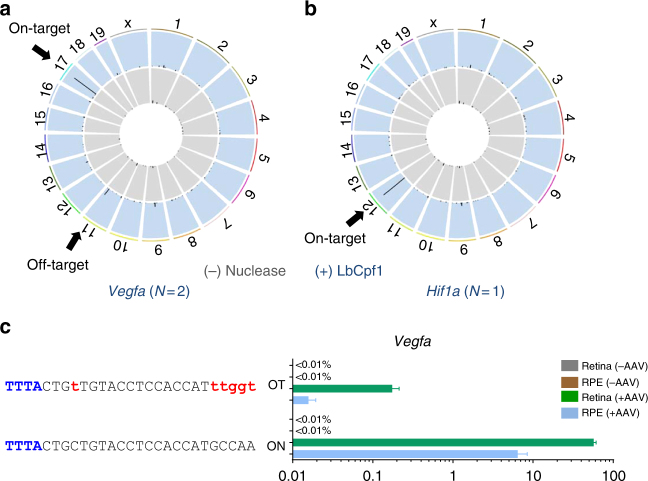
Retinal genome editing specificity of LbCpf1. **a**,**b** Mouse genomic DNA isolated from C57BL/6 J mice was digested in vitro by LbCpf1 and its crRNA targeted to *Vegfa* (**a**) or *Hif1a* (**b**) and subjected to whole-genome sequencing. Circos plots show genome-wide DNA cleavage scores across the mouse genome. Arrows indicate on-target or off-target sites. *N* indicates the number of in vitro cleavage sites identified by Digenome-seq. **c** Indel frequencies at in vitro cleavage sites identified by Digenome-seq. Genomic DNA isolated from retina and RPE cells 6 weeks after injection of AAV-Cpf1-*Vegfa* was subjected to targeted deep sequencing. Mismatched nucleotides are shown in red and PAM sequences in blue. ON, on-target site; OT, off-target site. Error bar indicates s.e.m. (*n* = 4)

We next investigated whether LbCpf1 has off-target nuclease activity in the mouse eye. To determine the genome-wide specificity of the *Vegfa*-or *Hif1a*-targeting LbCpf1 nuclease, we first carried out nuclease-digested whole genome sequencing (Digenome-seq). Cell-free mouse genomic DNA was digested in vitro using the *Vegfa*- or *Hif1a*-targeting LbCpf1 ribonucleoprotein (RNP) complex and then subjected to whole genome sequencing (WGS) (Fig. 2). The *Vegfa*- or *Hif1a*-targeting LbCpf1 cleaved two sites or one site, respectively, including the on-target site, in the mouse genome (Fig. 2a, b; Supplementary Table 2). These results are in line with previous reports showing the high specificity of Cpf1 in the human or mouse genome^22–24^. Next, we performed targeted deep sequencing at the Digenome-seq captured off-target site using genomic DNA isolated from AAV-Cpf1-*Vegfa* edited mouse retina and RPE. No off-target indels were detectably induced in RPE (Fig. 2c; Supplementary Table 3). In the Cpf1 edited retina, off-target indels were detected with a frequency of 0.17 ± 0.02%. We additionally analyzed 1 potential off-target site in the mouse genome, which differed from the *Vegfa* or *Hif1a* on-target site by up to four nucleotides in AAV-Cpf1-*Vegfa* or *-Hif1a* treated retina and RPE. Potential sites were identified using the Cas-OFFinder program. No off-target indels were detectably induced in AAV-Cpf1-*Vegfa* or *-Hif1a* injected mouse retina or RPE cells (Supplementary Fig. 6; Supplementary Table 4). Taken together, these results show that the LbCpf1 nuclease is targeted to *Vegfa* or *Hif1a* in the mouse retina in a highly specific manner in vivo.

### Therapeutic genome editing for the treatment of CNV

**Fig. 3:**
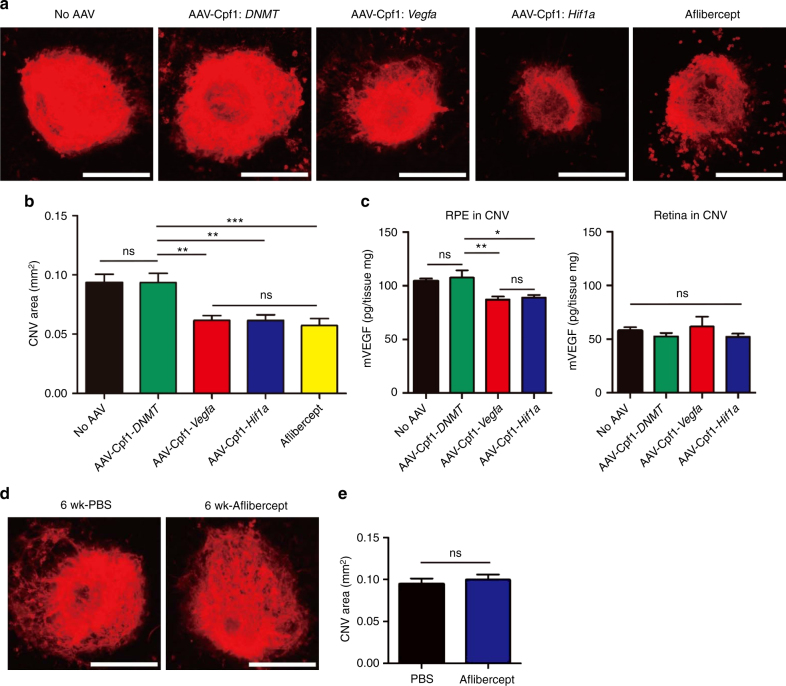
LbCpf1 targeted to *Vegfa* or *Hif1a* reduces the area of laser-induced CNV in mice. **a–c** At day 42 post-injection of AAV-Cpf1, mice were treated with laser to induce CNV. Aflibercept (2.5 μg) was intravitreally injected immediately after laser treatment. No AAV indicates no injection of AAV or aflibercept as a control. Three days after laser treatment, Vegfa protein levels were measured by ELISA in the retina and RPE complex. One week after laser treatment, the CNV area was analyzed. **a** Representative laser-induced CNV stained with isolectin B4 in mouse eyes injected with AAV-Cpf1 targeted to *DNMT*, *Vegfa*, or *Hif1a*. Scale bar is 200 μm. **b** The CNV area. Error bars indicate s.e.m. (*n* = 20). One-way ANOVA and Tukey’s *post-hoc* tests, * *P* < 0.05, ** *P* < 0.01, *** *P* < 0.001, ns, not significant. **c** VEGFA protein levels. Error bars indicate s.e.m. (*n* = 10). One-way ANOVA and Tukey’s *post-hoc* tests. *, *P* < 0.05; **, *P* < 0.01; ns not significant. **d–e** Six weeks after intravitreal injection of PBS or aflibercept (2.5 μg), mice were treated with laser to induce CNV. One week after laser treatment, the CNV area was analyzed. **d** Representative laser-induced CNV stained with isolectin B4 in mouse eyes injected with PBS or aflibercept. Scale bar is 200 μm. **e** The CNV area. Error bars indicate s.e.m. (*n* = 30). Student’s *t-*test. ns, not significant

Next, we induced CNV in the eye by laser treatment 6 weeks after injection of AAV-Cpf1-*Vegfa* or -*Hif1a* and measured the area of CNV^25^ 1 week later (Fig. 3a). AAV-Cpf1-*Vegfa* or -*Hif1a* reduced the area of CNV by 42 ± 4% and 34 ± 5%, respectively, compared to the AAV-uninjected negative control (Fig. 3b). A *DNMT*-specific Cpf1^22^, used as another negative control, which expresses crRNA but does not induce double strand breaks, also did not show any therapeutic effect. Additionally, we found that AAV-Cpf1-*Vegfa* or -*Hif1a* reduced VEGFA protein levels in the RPE by 17 ± 3 pg/mg and 15 ± 2 pg/mg, respectively, compared to the AAV-uninjected negative control (Fig. 3c). In contrast, the VEGFA protein levels in the retina were not significantly different among groups (Fig. 3c). These data imply that the decrease in the level of VEGFAprotein in the RPE might be linked to the decrease in the CNV area. When aflibercept, a widely-used anti-VEGF drug, was injected the same day as the laser treatment, it reduced the area of CNV by 39 ± 6% relative to the AAV-uninjected negative control, comparable to the therapeutic effect observed with injection of AAV-Cpf1-*Vegfa* or -*Hif1a* (Fig. 3a, b). However, when aflibercept was injected 6 weeks before the laser treatment, the area of CNV was not reduced (Fig. 3d, e), in line with the short half-life of the drug^26^. This result suggests that genome editing has a long-term therapeutic effect, whereas aflibercept must be injected multiple times to maintain its therapeutic effect on CNV.

### No retinal dysfunction after injection of AAV-Cpf1-*Vegfa* or -*Hif1a*

**Fig. 4:**
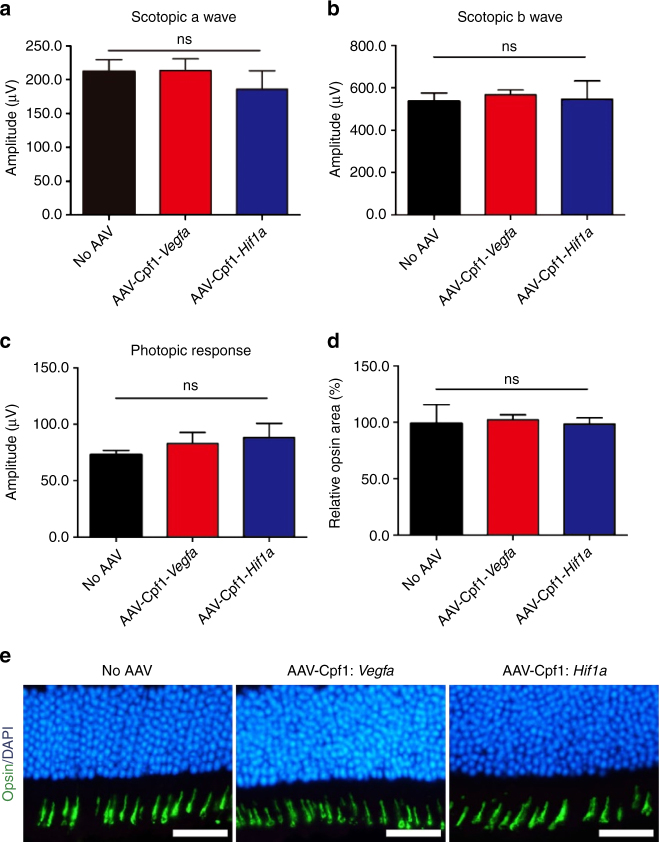
LbCpf1 targeted to *Vegfa* or *Hif1a*  does not affect cone function. **a–c** At day 42 post-AAV injection, full-field ERG was performed to evaluate retinal function. There was no significant decrease in the **a** scotopic a wave, **b** scotopic b wave, or **c** photopic response in mice treated with AAV-Cpf1-*Vegfa* or -*Hif1a* compared to normal control mice. Error bars indicate s.e.m. (*n* = 4). One-way ANOVA. ns, not significant. **d**, **e** Opsin-positive areas in the retina at day 42 post-injection. **d** Relative opsin-positive areas of the AAV-Cpf1-injected mice were normalized to that of the AAV-uninjected negative control mice. Error bars indicate s.e.m. (*n* = 4). Student’s *t-*test. ns not significant. **e** Representative images of opsin positive areas in mice treated with AAV-Cpf1-*Vegfa* or -*Hif1a* compared to the AAV-uninjected negative control mice (which received no AAV). Scale bar 20 μm

The conditional knockout of the *Vegfa* gene in mouse RPE cells leads to cone dysfunction^27^. To evaluate the potential toxicity of AAV-Cpf1-mediated *Vegfa* or *Hif1a* gene disruption, we first performed full-field electroretinography (ERG) in mice at 6 weeks after the intravitreal injection of AAV-Cpf1-*Vegfa* or *-Hif1a*. We observed no significant decrease in the scotopic response in these mice compared to that in untreated mice (Fig. 4a, b; Supplementary Fig. 7). In addition, there was no significant change in the photopic response (Fig. 4c; Supplementary Fig. 7). Then, we evaluated the level of opsin expression in the retina at 6 months after the intravitreal injection of AAV-Cpf1-*Vegfa* or *-Hif1a*. We also found that AAV-Cpf1-*Vegfa* or -*Hif1a* did not affect the size of the opsin-positive area in the retina, which is closely related to cone function (Fig. 4d, e), suggesting that AAV-Cpf1-mediated *Vegfa* or *Hif1a* gene disruption provides a safe therapeutic window.

## Discussion

In this report, we have shown that LbCpf1 can be successfully delivered to the mouse retina via an AAV serotype 9 vector and that it can induce efficient gene editing in the retina and RPE cells. LbCpf1 induced indels at high frequencies in the *Vegfa* and *Hif1a* genes, suggesting its potential for the treatment of angiogenesis-associated diseases. Cpf1 has several benefits for therapeutic applications. First, Cpf1 is highly specific in human cells with minimum off-target activities, compared to SpCas9 as shown in previous reports^22,23^. The use of Cpf1 also allows for streamlined multiplex genome editing. Because Cpf1 can process pre-crRNA arrays, multiple pre-crRNAs can be expressed from a single U6 promoter without the need for additional sequences^19,28^.

In vivo therapeutic genome editing is an active area of research. However, unlike ex vivo approaches, in vivo genome editing is limited by potential immune responses against Cas9 or Cpf1 and relatively poor efficiency of transgene delivery. In this regard, the eye is an ideal organ for in vivo therapeutic editing because it is immune-privileged. AAV vectors are powerful tools for delivering the genome engineering machinery to the ocular tissues^29–31^. Indeed, a number of studies have demonstrated efficient expression of transgenes using AAV serotype 9 in the mouse retina^32–35^. In our previous study, we also showed that Cas9 derived from *Campylobacter jejuni* is efficiently expressed in the retina and RPE cells after intravitreal injection via an AAV9 vector^35^. In this study, we used AAV serotype 9 as a vehicle to deliver Cpf1 and crRNA into mouse retina. In contrast to AAV serotype 2, which targets RPE cells only when delivered by subretinal injection, AAV9 was efficiently transfected into RPE cells after intravitreal injection^35^.

AAV-mediated genome editing may cause systemic side effects if AAV passes through the bloodstream during intravitreal injection. However, several reports support the safety of AAV in the treatment of retinal diseases. In LCA clinical trials, there was no evidence of systemic dissemination of AAV vectors in tears, serum, or saliva samples 30 days after subretinal administration of AAV2/2-hRPE65^36–38^. Sugano et al. and Shih et al. also assessed the presence of AAV following intravitreal injection: AAV was detected within the ocular tissues but not in other organs^39,40^. However, a study of the immune response to Cpf1 should also be investigated.

When delivered to the retina and RPE, AAV-Cpf1-*Vegfa* or *Hif1a* induced *Vegfa* or *Hif1a* gene disruption, respectively followed by reduction of the CNV area in a mouse model of AMD. Its anti-angiogenic effect was comparable to that of aflibercept. Note, however, that unlike AAV-Cpf1-*Vegfa* or *Hif1a*, aflibercept did not inhibit neovascularization and did not reduce the area of CNV when given 6 weeks before the laser treatment, confirming its short half-life in vivo^26^. This finding suggests that AAV-LbCpf1 has a long-term therapeutic effect even with a single administration and could replace anti-VEGF drugs currently in use in the clinic. Note that aflibercept and other anti-VEGF drugs must be administered repetitively, often for a lifetime. Evidence of long-term expression of sequences delivered by AAV is supported by several promising, ongoing clinical trials using AAV vectors for retinal disorders^41^. It has also been reported that AAV-mediated transgene expression continued in non-human primates for as long as 5.5 years after gene transfer without any detectable toxicity^42^. The ability of AAV vectors to mediate long-term transgene expression offers a means of treating life-long chronic diseases such as AMD with a single administration of vector. As such, the use of AAV-Cpf1 may reduce the cost burden of treatment for patients and improve the quality of life. Our results support that application of AAV-LbCpf1 as a gene editing tool will be an effective strategy for treating AMD and other diseases associated with neovascularization.

## Methods

### Construction of an AAV vector plasmid encoding LbCpf1 and crRNA

A human codon-optimized LbCpf1 coding sequence, derived from *Lachnospiraceae bacterium* ND 2006, was purchased from Addgene (plasmid # 78744). The sequence was cloned into an AAV inverted terminal repeat (ITR)-based vector plasmid. The *Vegfa*, *HIf1a*, or *DNMT* targeting crRNA sequence (Supplementary Table 1) was also inserted to create pAAV-ITR-LbCpf1-crRNA. crRNAs were transcribed under the control of the U6 promoter and LbCpf1 expression was controlled by the elongation factor-1 alpha short (EFS) promoter in C2C12 (ATCC, CRL-1772) cells and in the mouse retina and RPE.

### Cell culture and mutation analysis

Cells were maintained in Dulbecco’s Modified Eagle’s Medium (DMEM, Welgene, cat. no. LM001-05) supplemented with 100 units per ml penicillin (Gibco, cat. no. 15140–122), 100 μg/ml streptomycin, and 10% fetal bovine serum heat-inactivated (FBS, Welgene, cat. no. S 101–01). In this study, we investigated the efficacy of several crRNAs in C2C12 cells because these cells are efficiently transfected. Cells (1 × 10^5^) were seeded into 24-well plates one day prior to transfection and transfected with the crRNA plasmid (1500 ng) and the Cpf1 plasmid (500 ng) using 4 μl of Lipofectamine 2000 (Invitrogen, cat. no. 11668019). Cells were maintained in DMEM supplemented with 2% FBS for differentiation. Genomic DNA was isolated using a DNeasy Blood & Tissue kit (Qiagen, cat. no. 69581) 48 h post-transfection. On-target or off-target loci were amplified using 100 ng of genomic DNA for targeted deep sequencing. Deep-sequencing libraries were generated by PCR. TruSeq HT Dual Index primers were used to label each sample. Pooled libraries were subjected to paired-end sequencing using MiniSeq (Illumina). Indel frequencies are described in Supplementary Table 1.

### Production and titration of AAV vectors

To produce AAV vectors, they were pseudotyped in AAV9 capsids. HEK293T cells (ATCC, CRL-3216) were transfected with pAAV-ITR-LbCpf1-crRNA, pAAV2/9 encoding for AAV2rep and AAV9cap, and helper plasmid. HEK293T cells were cultured in DMEM with 2% FBS. Recombinant pseudotyped AAV vector stocks were generated using PEI coprecipitation with PEIpro (Polyplus-transfection) and triple-transfection with plasmids at a molar ratio of 1:1:1 in HEK293T cells. After 72 h of incubation, cells were lysed and particles were purified by iodixanol (Sigma-Aldrich) step-gradient ultracentrifugation. The number of vector genomes was determined by quantitative PCR.

### Digenome sequencing

Genomic DNA was isolated from liver tissue of C57BL/6 mice using a DNeasy Blood & Tissue kit (Qiagen) according to the manufacturer’s instructions. Genomic DNA (8 μg) was mixed with LbCpf1 protein (300 nM) and crRNA (900 nM) in a 400 μl reaction buffer (100 mM NaCl, 50 mM Tris-HCl, 10 mM MgCl2, and 100 μg/ml BSA) and the mixture was incubated for 8 h at 37 °C. Digested genomic DNA was then incubated with RNase A (50 μg/ml) for 30 min at 37 °C to degrade crRNAs and purified again with a DNeasy Blood & Tissue kit (Qiagen). Digested DNA was fragmented using the Covaris system and ligated with adapters for library formation. DNA libraries were subjected to whole-genome sequencing using an Illumina HiSeq X Ten Sequencer at Macrogen (South Korea)^43,44^. We used the Isaac aligner to generate a Bam file using the following parameters: ver. 01.14.03.12; Mouse genome reference, mm10 from UCSC; Base quality cutoff, 15; Keep duplicate reads, yes; Variable read length support, yes; Realign gaps, no; and Adapter clipping, yes (adapter: AGATCGGAAGAGC*, *GCTCTTCCGATCT)^45^. A DNA cleavage score was assigned to each nucleotide position across the entire genome, using WGS data, according to the equation presented in Kim et al^22^. These equations assume that Cpf1 produces 5′ 1- to 5-nt overhangs. In vitro cleavage sites with DNA cleavage scores above the cut-off value of 2.5 were computationally identified.

### Animals

The care, use, and treatment of all animals in this study were in strict agreement with the ARVO statement for the Use of Animals in Ophthalmic and Vision Research, the College of Veterinary Medicine guidelines, and the guidelines established by the Seoul National University Institutional Animal Care and Use Committee, which granted permission to perform animal experiments. Eight-week-old, male, specific pathogen-free C57BL/6 J mice (*n* = 4–9) were used in this study. Mice were maintained under a 12 h dark-light cycle.

### Laser-induced CNV model

After mice were anesthetized, pupils were dilated with an eye drop containing phenylephrine (0.5%) and tropicamide (0.5%). Laser photocoagulation was performed using an indirect head set delivery system (Iridex) and laser system (Ilooda). Laser parameters were 810 nm wave length, 200 μm spot size, 800 mW power, and 70 ms exposure time. Laser burn was induced 3–4 times around the optic disc. Only burns that produced a bubble without vitreous hemorrhage were included in the study. Seven days later, the eyes were fixed in 4% paraformaldehyde for 1 h at room temperature. RPE complexes (RPE/choroid/sclera) were prepared for immunostaining and then incubated with isolectin-B4 (Thermo Fisher Scientific, cat. no. I21413, 1:100) overnight at 4 °C. The RPE complex was flat-mounted and viewed with a fluorescent microscope (Eclipse 90i, Nikon) or a confocal microscope (LSM 710, Carl Zeiss) at a magnification of 100×. The CNV area was measured using Image J software (1.47 v, NIH) by blinded observers. An average of 3–4 CNV areas per eye were analyzed.

### Intravitreal injection of AAV

Eight-week-old mice were anesthetized with an intraperitoneal injection of a mixture of tiletamine and zolazepam (1:1, 2.25 mg/kg body weight) and xylazine hydrochloride (0.7 mg/kg body weight). AAV2/9-LbCpf1-*Vegfa* or *-Hif1a* (2 × 10^10^ viral genomes in 2 μl) was intravitreally injected using a Nanofil syringe with a 33 G blunt needle (World Precision Instruments Inc.) under an operating microscope (Leica Microsystems Ltd.). The virus dose used in this study was limited due to production issues, and higher virus doses may elicit higher mutation efficacy.

### Immunofluorescent staining and imaging of retinal tissue

For the analysis of the opsin-positive area, formalin-fixed paraffin embedded samples were prepared at day 42 post-injection (*n* = 4). Cross-section samples were immunostained with anti-opsin antibody (Millipore, cat. no. AB5405, 1:1000) and Alexa Fluor 488 antibody (Thermo Fisher Scientific, cat. no. A-11034, 1:500). The opsin-positive area was measured using Image J software (1.47 v, NIH) by blinded observers. To visualize the distribution of HA-tagged Cpf1, the eyes were fixed in 4% paraformaldehyde for 1 h at room temperature. RPE complexes (RPE/choroid/sclera) were prepared for immunostaining and then incubated with anti-HA antibody (Roche, cat. no. 3F10, 1:100) overnight at 4 °C. After staining with Alexa Fluor 594 antibodies (Thermo Fisher Scientific, cat. no. A-11006, 1:500), the RPE flat-mounts were imaged using a confocal microscope (LSM 710, Carl Zeiss). The scanning parameters were as follows: scaling (*x* = 0.042 μm/pixel, *y* = 0.042 μm/pixel, *z* = 0.603 μm/pixel), dimensions (*x* = 1024, *y* = 1024, channels: 2, 8-bit) with objective C-Apochromat 40 × /1.20 W Korr M27. ZEN 2 software was used to process the images.

### Mouse VEGFA ELISA

At day 42 post-injection of AAV2/9-LbCpf1, mice were treated with laser. Three days after laser treatment, whole RPE complexes were separated from neural retina tissue and frozen for further analysis. Sample tissues were lysed in RIPA buffer (120 μl) and Vegfa protein levels were measured using a mouse VEGF Quantikine ELISA kit (R&D systems, cat. no. MMV00) according to the manufacturer’s instructions.

### Electroretinography (ERG) analysis

Mice were dark-adapted over 16 h. Mice were anesthetized with an intraperitoneal injection of a mixture of tiletamine and zolazepam (1:1, 2.25 mg/kg body weight) and xylazine hydrochloride (0.7 mg/kg body weight). Pupils were dilated with an eye drop containing phenylephrine (0.5%) and tropicamide (0.5%). Contact lens electrodes were placed on both eyes with a drop of methylcellulose. Full-field ERGs were recorded^46^ by using the universal testing and electrophysiologic system 2000 (UTAS E-2000, LKC Technologies). The responses were recorded at a gain of 2 k using a notch filter at 60 Hz, and were bandpass filtered between 0.1 and 1500 Hz. In the light-adapted photopic state, with a 30 cd/m^2^ background light to desensitize the rods and isolate cones, photopic cone responses were recorded in response to a single flash of 0 dB. The amplitude of the a-wave was measured from the baseline to the lowest negative-going voltage, whereas peak b-wave amplitudes were measured from the trough of the a-wave to the highest peak of the positive b-wave.

### Statistical analysis

No statistical methods were used to predetermine sample size for in vitro or in vivo experiments. All group results are expressed as mean ± SEM, if not stated otherwise. Comparisons between groups were made using the two-tailed Student’s *t*-test or one-way ANOVA and Tukey *post-hoc* tests for multiple groups. Statistical significance as compared to untreated controls is denoted with * (*P* < 0.05), ** (*P* < 0.01), *** (*P* < 0.001) in the figures and figure legends. Statistical analysis was performed in Graph Pad PRISM 5.

### Data availability

The deep sequencing data from this study have been submitted to the NCBI Sequence Read Archive under accession number SRP129908. The data that support the findings of this study are available from the corresponding author upon reasonable request.

## Electronic supplementary material

Supplementary Information
