# Supplementary material for: CRISPR-LbCpf1 prevents choroidal neovascularization in a mouse model of age-related macular degeneration
Source: Nat Commun. 2018 May 10;9:1855. doi: 10.1038/s41467-018-04175-y (PMC5945874; doi:10.1038/s41467-018-04175-y)
Supplement: Supplementary file 1 — Supplementary Information [file 41467_2018_4175_MOESM1_ESM.pdf]

Supplementary Information for Koo *et al.*

CRISPR-LbCpf1 prevents choroidal neovascularization in a mouse model of age-related macular degeneration

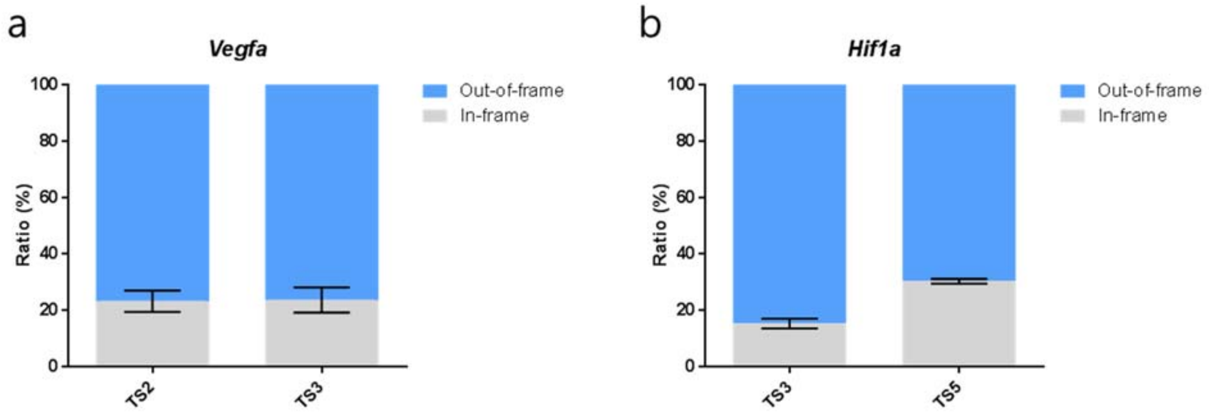

Supplementary Figure 1. Mutant sequences presented with two fractions, in-frame versus out-of-frame indels at the *Vegfa* (a) or *Hif1a* (b) target site. crRNA plasmids were transfected into mouse C2C12 cells together with LbCpf1 plasmids. Mutation sequences were analyzed by deep sequencing using genome DNA isolated from cells after 48 h of transfection. Mutant sequences are divided into two fractions, out-of-frame versus in-frame indels and presented in ratio (%). Error bars indicate s.e.m. (n=3).

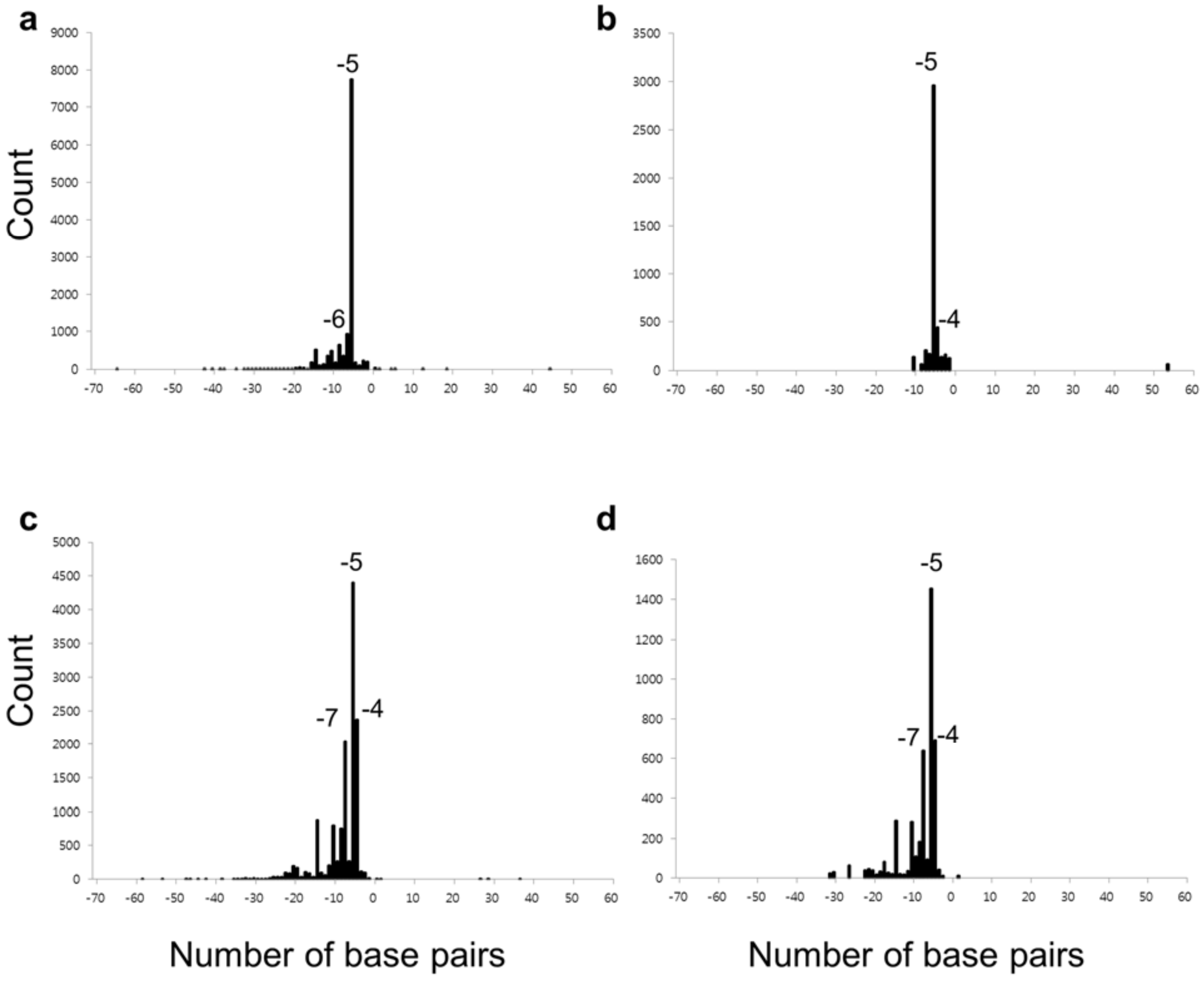

Supplementary Figure 2. The number of mutant sequence reads binned by the deletion or insertion size in base pairs at the *Vegfa* (a, b) and *Hif1a* (c, d) target sites in the retina (a, c) and RPE (b, d) cells using deep sequencing 6 weeks post-intravitreal injection of AAV9-Cpf1-*Vegfa or Hif1a*.

Retina RPE

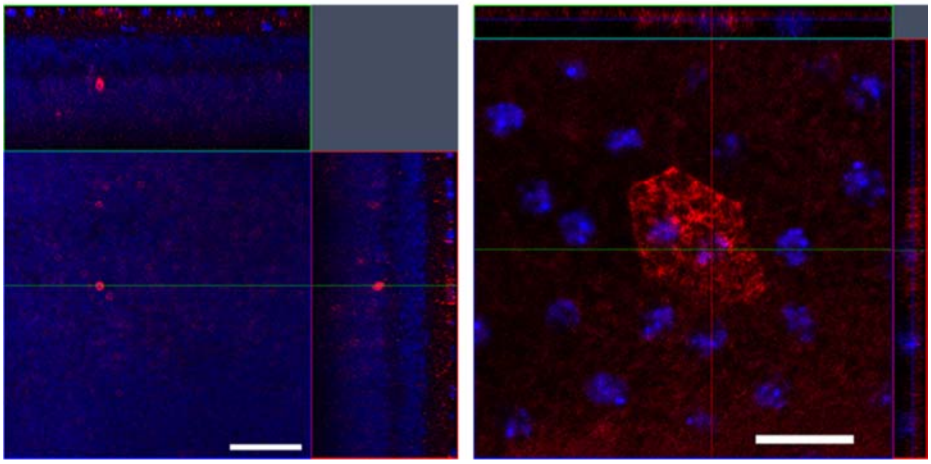

Supplementary Figure. 3. Representative confocal images of *in vivo* HA expression in retina (x 400, scale bar, 50 μm) and RPE cells (x 1000, scale bar, 20 μm) of AAV-Cpf1-*Hif1a* injected mice 6 weeks after injection (n=4). HA was stained with anti-HA antibody (red). Nuclei were counter-stained with DAPI (blue).

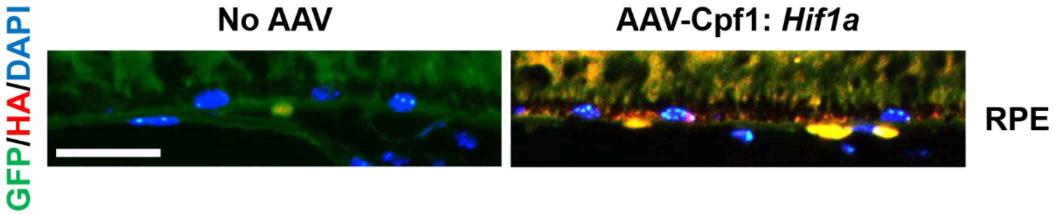

Supplementary Figure. 4. Expression of GFP and HA in the retinal pigment epithelium of mice treated with PBS (No AAV) or AAV-Cpf1-*Hif1a*. Scale bar, 20 μm. RPE, retinal pigment epithelium.

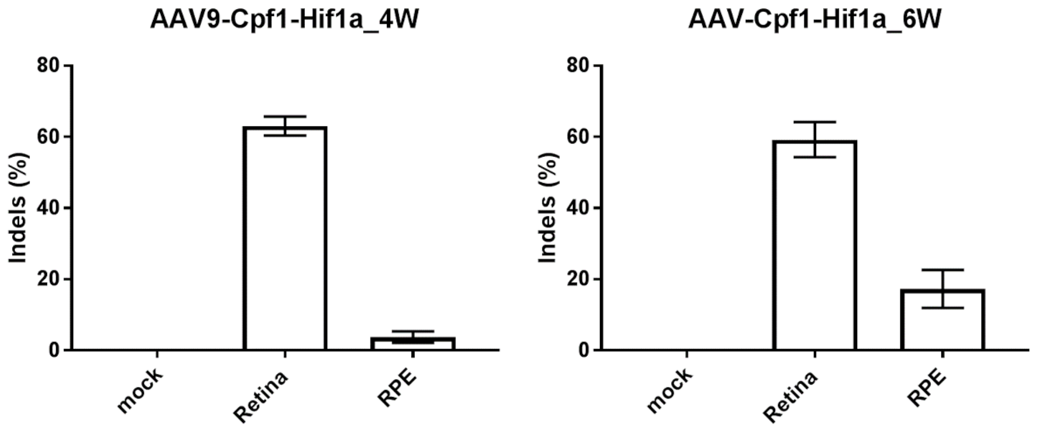

Supplementary Figure. 5. Retinal genome editing of LbCpf1. Indel frequencies at the *Hif1a* target site in the retina and RPE cells using deep sequencing 4 and 6 weeks post-intravitreal injection of AAV9-Cpf1-*Hif1a*. Error bar indicates s.e.m. (n = 4).

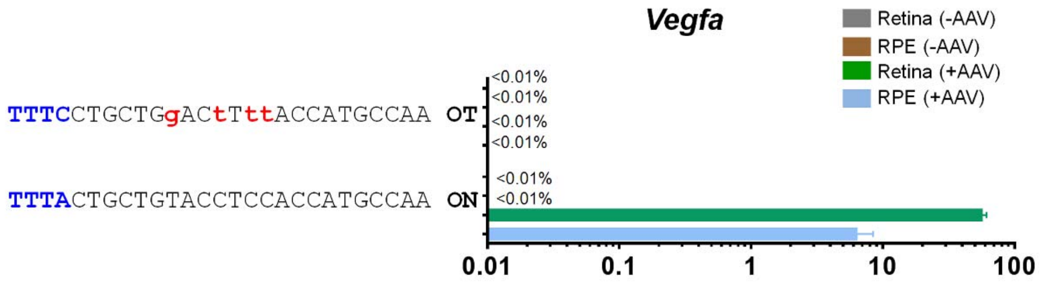

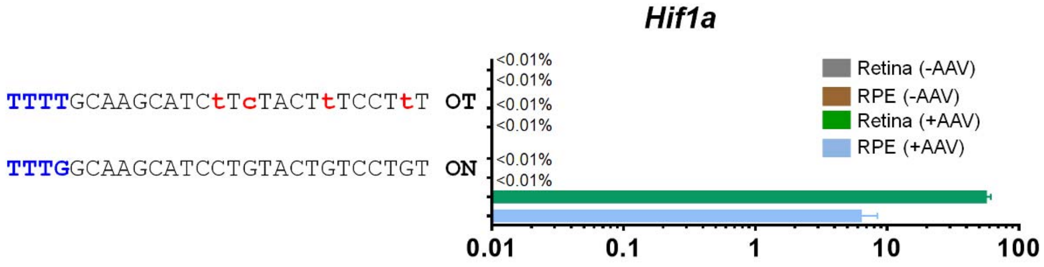

Supplementary Figure. 6. Indel frequencies at on-target and potential off-target predicted by Cas-OFFinder. No off-target indels were detected at the homologous site, which differed from the on-target sites by up to 4 nucleotides, in the mouse genome. Mismatched nucleotides are shown in red and PAM sequences in blue. ON; on-target site, OT; off-target site. Error bar indicates s.e.m. (n = 4).

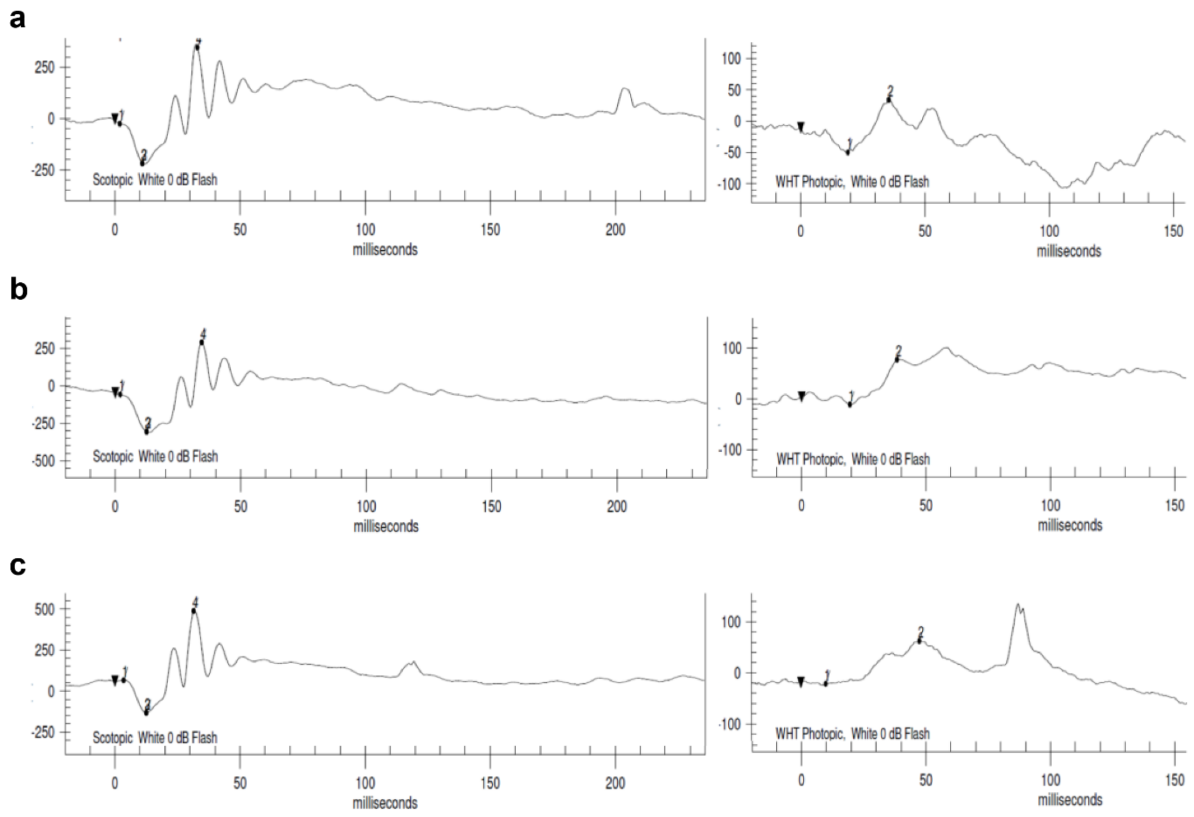

Supplementary Figure. 7. Full-field ERG in mice treated with AAV-Cpf1-*Vegfa* or -*Hif1a* compared to normal control mice. (a) Representative scotopic (left) and photopic (right) responses of normal control mice. (b) Representative scotopic (left) and photopic (right) responses of mice treated with AAV-Cpf1-*Vegfa*. (c) Representative scotopic (left) and photopic (right) responses of mice treated with AAV-Cpf1-*Hif1a*.

Supplementary Table 1. LbCpf1 crRNA list used in this study.

#	Target site	crRNA	Target sequences (5' to 3')	PAM (5' to 3')	Indels* (%)	s.e.m (%)
1	mouse *Vegfa*	Vegfa-TS1	GGAGGCCCGTCCGGGGCCGGCGC	TTTC	1.7	0.20
2		Vegfa-TS2	TGCTCTCTTGGGTGCACTGGACC	TTTC	13.7	1.34
3		Vegfa-TS3	CTGCTGTACCTCCACCATGCCAA	TTTA	19.2	1.35
4		Vegfa-TS4	GTGAGGTTTGATCCGCATGATCT	TTTG	12.9	0.85
5		Vegfa-TS5	TTCTGTCTTTCTTTGGTCTGCAG	TTTG	5.1	0.38
6		Vegfa-TS6	TGGCTTTGTTCTGTCTTTCTTTG	TTTC	6.4	0.31
7		Vegfa-TS7	CTGGCTTTGTTCTGTCTTTCTTT	TTTT	1.1	0.14
8		Vegfa-TS8	TCTACCCACTGCAGACCAAAGAA	TTTC	12.1	0.72
9	Mouse *Hif1a*	Hif1a-TS1	ACTCATCCATGTGACCATGAGGA	TTTT	1.5	0.08
10		Hif1a-TS2	CTAAAGGACAAGTCACCACAGGA	TTTA	16.3	0.87
11		Hif1a-TS3	GCAAGCATCCTGTACTGTCCTGT	TTTG	12.2	0.24
12		Hif1a-TS4	GGCAAGCATCCTGTACTGTCCTG	TTTT	4.7	1.03
13		Hif1a-TS5	AACCCAGACATATCCACCTCTTT	TTTC	24.5	0.36
14		Hif1a-TS6	TTGAAGGGAGAAAATCAAGTCGT	TTTG	21.7	0.63
15		Hif1a-TS7	GACAGTGGTATTATTCAGCACGA	TTTT	10.9	0.35
16		Hif1a-TS8	ACAGTGGTATTATTCAGCACGAC	TTTG	16.4	0.47
17	Human *DNMT*	DNMT-TS1	CTGATGGTCCATGTCTGTTACTC	TTTC	N/A	N/A

*Indel frequencies (%) in Fig.1a.

Supplementary Table 2. Indel frequencies at on-target and potential off-target validated by Digenome-seq. Mismatched nucleotides are shown in blue and PAM sequences in red.

Target	Location			Target sequence (5' to 3')	Cleavage score
Vegfa-TS3	On-target	Chr17	46031291	TTTACTGCTGTACCTCCACCATGCCAA	22.4
	Off-target	Chr11	105284128	TTTACTGtTGTACCTCCACCATttggt	6.2
Hif1a-TS3	On-target	Chr12	73937675	TTTGGCAAGCATCCTGTACTGTCCTGT	23.2

Supplementary Table 3. Indel frequencies at on-target and potential off-target validated by Digenome-seq. Mismatched nucleotides are shown in blue and PAM sequences in red.

Target		Location	Target sequence (5' to 3')	Indel frequency (%)*		Indel frequency (%)**	
				(-) RGEN	(+) RGEN	(-) RGEN	(+) RGEN
Vegfa-TS3	On-target	Chr17	**TTTA**CTGCTGTACCTCCACCATGCCAA	0.00	6.45	0.00	57.17
	Vegfa-TS3-OT1	Chr11	**TTTA**CTG**t**TGTACCTCCACCAT**ttggt**	0.003	0.02	0.00	0.17

RPE*, Retina**

Supplementary Table 4. Indel frequencies at on-target and potential off-target predicted by Cas-OFFinder. Mismatched nucleotides are shown in blue and PAM sequences in red.

Target		Location	Target sequence (5' to 3')	Indel frequency (%)*		Indel frequency (%)**	
				(-) RGEN	(+) RGEN	(-) RGEN	(+) RGEN
Vegfa-TS3	On-target	Chr17	**TTTA**CTGCTGTACCTCCACCATGCCAA	0.00	6.45	0.00	57.17
	Vegfa-TS3-OT1	Chr10	**TTTC**CTGCTG**g**AC**t**T**tt**ACCATGCCAA	0.00	0.01	0.007	0.00
Hif1a-TS3	On-target	Chr12	**TTTG**GCAAGCATCCTGTACTGTCCTGT	0.00	17.23	0.00	59.21
	Hif1a-TS3-OT1	Chr14	**TTTT**GCAAGCATC**t**T**c**TACT**t**TCCT**t**T	0.00	0.01	0.00	0.01

RPE*, Retina**
